# Rezafungin Utilisation in Real Life—FungiScope Results From Europe and the United States

**DOI:** 10.1111/myc.70114

**Published:** 2025-09-17

**Authors:** Ilana Reinhold, Giovanni Mori, Massimiliano Lanzafame, Alessandro Limongelli, Antonio Vena, Julia Götz, Stilla Bauernfeind, Frank Hanses, Lukas Tometten, Michael Mayer, Ansgar Rieke, Ana Soriano‐Martin, Maricela Valerio, Jose A. Vazquez, Patrick Yue, Laman Rahimli, Nijat Azimli, Ertan Sal, Jon Salmanton‐García, Natalia Vasenda, Rosanne Sprute, Jannik Stemler, Sebastian Wingen‐Heimann, Oliver A. Cornely, Danila Seidel

**Affiliations:** ^1^ Institute of Translational Research, Cologne Excellence Cluster on Cellular Stress Responses in Aging‐Associated Diseases (CECAD), Faculty of Medicine University of Cologne Cologne Germany; ^2^ Department I of Internal Medicine, Center for Integrated Oncology Aachen Bonn Cologne Duesseldorf (CIO ABCD) and Excellence Center for Medical Mycology (ECMM), Faculty of Medicine University of Cologne, University Hospital Cologne Cologne Germany; ^3^ German Centre for Infection Research (DZIF), Partner Site Bonn‐Cologne Cologne Germany; ^4^ Unit of Infectious Diseases, Santa Chiara Hospital, Azienda Provinciale per i Servizi Sanitari Trento Italy; ^5^ Centre for Medical Sciences (CISMed) University of Trento Trento Italy; ^6^ Division of Infectious Diseases, Department of Health Sciences (DISSAL) University of Genova Genova Italy; ^7^ IRCCS Ospedale Policlinico San Martino Genova Italy; ^8^ Department I of Internal Medicine, Division of Infectious Diseases University of Cologne, Faculty of Medicine, University Clinics Cologne Germany; ^9^ Universitätsklinikum Regensburg, Abt. Krankenhaushygiene und Infektiologie Regensburg Germany; ^10^ Department of Internal Medicine Gemeinschaftsklinikum Mittelrhein Koblenz Germany; ^11^ Clinical Microbiology and Infectious Diseases Department Hospital General Universitario Gregorio Marañón Madrid Spain; ^12^ Instituto de Investigación Sanitaria Gregorio Marañon Madrid Spain; ^13^ Departamento de Medicina Universidad Complutense Madrid Spain; ^14^ CIBER Enfermedades Respiratorias (CIBERES), Instituto de Salud Carlos III Madrid Spain; ^15^ Department of Medicine/Division of Infectious Disease Medical College of Georgia/Augusta University Augusta Georgia USA; ^16^ Faculty of Medicine and University Hospital Cologne, Clinical Trials Centre Cologne (ZKS Köln) University of Cologne Cologne Germany

**Keywords:** antifungal treatment, candidemia, chronic pulmonary aspergillosis, invasive candidiasis

## Abstract

**Background:**

Rezafungin, a novel echinocandin with once‐weekly intravenous dosing, offers potential advantages for outpatient parenteral antifungal therapy (OPAT) in invasive candidiasis (IC). While clinical trial data support its efficacy and safety, real‐world experience remains limited.

**Methods:**

A retrospective analysis of patients treated with rezafungin across Germany, Italy, Spain, and the United States between January 2024 and June 2025 was conducted. Data was collected via the FungiScope registry. Clinical characteristics, indications for rezafungin, outcomes, safety, and logistical aspects of administration were evaluated.

**Results:**

Fifteen patients were included, fourteen with IC; one with chronic pulmonary aspergillosis. Regarding patients with IC, the median age was 65.5 years; 43% were female. The most frequently identified pathogens were *Candida glabrata* (57%) and *Candida parapsilosis* (21%). Primary indications for rezafungin were intravascular (36%) and osteoarticular infections (36%). Rezafungin was mainly selected to enable OPAT (86%) or due to fluconazole resistance (36%) or drug–drug interactions (14%). The median treatment duration was 9 weeks (range: 1–38 weeks). One mild adverse event occurred (cutaneous photosensitivity), but rezafungin was otherwise well tolerated. Complete clinical or mycological response was observed in 36% at day 30, and partial response in 50% of patients. Access differed substantially across centres due to administrative and reimbursement hurdles, affecting treatment transition to rezafungin in 71% of patients with IC.

**Conclusions:**

Rezafungin was effective and well tolerated in this cohort, particularly in patients requiring long‐term treatment. Administrative and logistical hurdles remain significant barriers to its widespread use. Facilitated access and enhanced awareness may improve patient outcomes by supporting early initiation and continuity of care.

## 1 Introduction

Invasive candidiasis (IC) and candidemia remain life‐threatening infections, particularly in critically ill and immunocompromised patients. Successful management has been complicated by the shift of *Candida albicans* to non‐*albicans* species over the past decade, including *Candida glabrata* (*Nakaseomyces glabratus*), *Candida parapsilosis* , and *Candida auris* [1]. Echinocandins remain the first‐line antifungal for IC, whereby treatment requires daily intravenous (i.v.) administration [2]. This limits usability in the outpatient setting and contributes to prolonged hospital stays [3]. This can be particularly challenging in IC associated with deep‐seated infections requiring antifungal treatment over several weeks or months [2].

Rezafungin, a novel echinocandin with a prolonged half‐life and a once‐weekly i.v. dosing, was approved for the treatment of IC and candidemia in adults by the Food and Drug Administration (FDA) in March 2023, and by the European Medicines Agency (EMA) in December 2023 [4]. Rezafungin exhibits in vitro activity against *Candida* spp. including azole‐resistant strains, *Aspergillus* spp. and *Pneumocystis* spp. [5, 6]. Approval was supported by a phase 3 multicentre study, demonstrating once‐weekly rezafungin was non‐inferior to once‐daily caspofungin in adults with IC or candidemia in terms of global cure rate at day 14 and all‐cause mortality at day 30 [7]. Maximum treatment duration in phase 2 and phase 3 trials was 28 days for rezafungin [7, 8]. However, there are no limits to longer treatment durations within the approval for rezafungin. Case reports from the early access programme with prolonged treatment demonstrated safety for prolonged treatment durations [9].

Since the licensing of rezafungin, real‐world data on its use has only recently begun to emerge. This multinational case series aims to describe the indications, safety, tolerability, and real‐world barriers to rezafungin application following its marketing authorisation in Europe and the USA. By identifying potential obstacles, we aim to inform clinical practice and support improved accessibility to rezafungin across different healthcare settings.

## 2 Methods

We retrospectively collected data on patients receiving rezafungin from the global FungiScope registry (ClinicalTrials.gov identifier: NCT01731353). FungiScope is an international, anonymised, web‐based registry of invasive fungal infections [10]. It is approved by the Institutional Review Board and Ethics Committee of the University Hospital Cologne, Germany (Study ID: 05‐102). This analysis was conducted as part of the Candida Campaign, a dedicated initiative within FungiScope. Patients treated with rezafungin between January 2024 and June 2025 were included in this analysis, including one patient with chronic pulmonary aspergillosis.

All data were entered into the registry by treating clinicians using an electronic case report form (clinicalsurveys.net, Tivian, Cologne, Germany). For each patient, detailed clinical information was collected, including patient demographics, underlying medical conditions, diagnostic methods, fungal species identification, antifungal susceptibility, indications for rezafungin use, prior antifungal therapies, clinical and mycological response, and outcome. Minimum inhibitory concentrations (MICs) were documented for anidulafungin and fluconazole or other antifungals where available. MIC values were reported according to the European Committee on Antimicrobial Susceptibility Testing (EUCAST) broth microdilution method in Germany and Italy (EUCAST E.Def 7.4) and Clinical and Laboratory Standards Institute (CLSI) broth microdilution method (CLSI M27‐A3 4th edition) in the USA and Spain. The submitted cases underwent a review by an infectious disease (ID) specialist to ensure data consistency and accuracy. Global response to antifungal treatment was defined according to clinical, radiological and mycological criteria and classified as complete response, partial response, stable response, progression and death [11].

Further objectives of the study were the feasibility of outpatient parenteral antimicrobial therapy (OPAT), the safety and tolerability profile of rezafungin, and challenges encountered before and during its administration.

Descriptive statistics were performed using Microsoft Excel (Microsoft Corp., 2021). Categorical variables are reported as frequencies and percentages, and continuous variables as mean, median, and range.

## 3 Patient Descriptions

**FIGURE 1:**
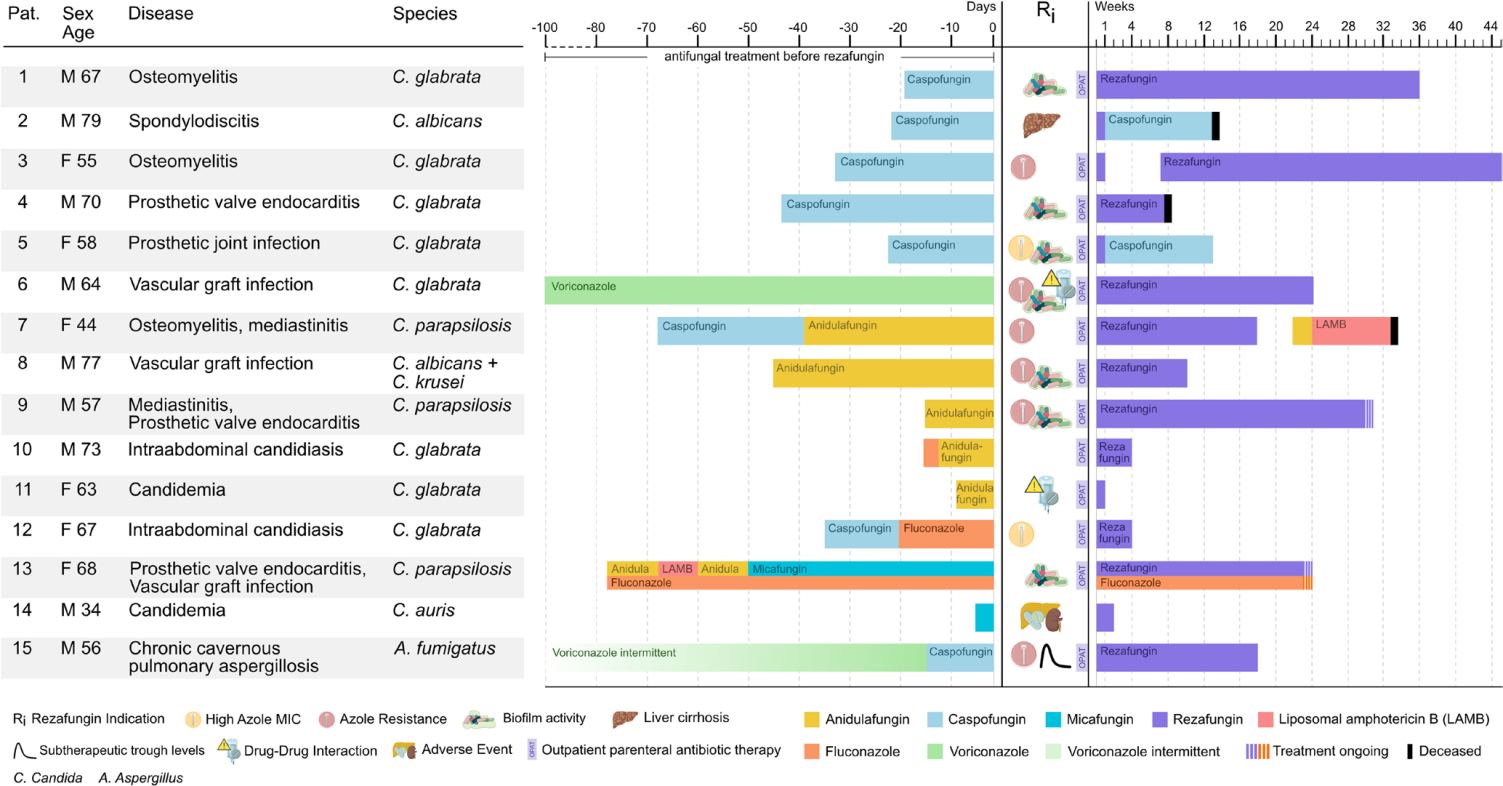
Indications for rezafungin administration in 15 patients, and antifungal therapies administered prior to and following rezafungin initiation (icons adapted from biorender.com).

All patients are summarised in Table 1. Figure 1 provides timelines of antifungal treatment strategies before and after rezafungin administration for each of the 15 patients.

**TABLE 1:** Patient overview of 15 patients treated with rezafungin.

	Patient 1	Patient 2	Patient 3	Patient 4	Patient 5
Age (years)	67	79	55	70	58
Sex	M	M	F	M	F
Country	Germany	Germany	Germany	Germany	Germany
Date of invasive fungal infection diagnosis	Mar 24	Apr 24	Aug 24	Oct 24	Jan 25
Medical history	CAD, CABG	Liver cirrhosis Child B, COPD	CAD, CABG, DM	CAD, TAVI, DM	Chronic osteomyelitis of the left mandibula
Fungal species	*C. glabrata*	*C. albicans*	*C. glabrata*	*C. glabrata*	*C. glabrata*
Diagnosis	*C. glabrata* and *Streptococcus mitis* sternal osteomyelitis	Multisegmented thoracic spondylodiscitis	*C. glabrata* and *Staphylococcus caprae* sternal osteomyelitis	Prosthetic aortic endocarditis	*C. glabrata, Enterobacter cloacae* and *Pseudomonas aeruginosa* temporomandibular joint prosthesis infection
MIC (μg/mL)	AND 0.03 FLU 4	AND 0.016 FLU 0.5	AND 0.06 FLU 128	AND 0.15 FLU 2	AND 0.012 FLU 8
Indication for rezafungin	OPAT, biofilm activity (cerclage removal refused)	Hepatopathy, OPAT intended	OPAT, azole resistance	OPAT, long‐term suppressive therapy (palliative care), biofilm activity	OPAT, echinocandin for prosthesis infection, conservative management
Prior antifungal treatment and duration	CAS 19 d	CAS 23d	CAS 33 d	CAS 44 d	CAS 22 d
Duration of rezafungin (weeks)	36	1	38	8	1
D30 Clinical/radiological/mycological response	Partial response	Partial response	Partial response	Progressive disease (recurrent candidemia d20 under CAS, d68 under rezafungin)	Progressive disease (necrotic wound and polymicrobial infection) requiring surgery
Day 31 to 180 Clinical/radiological/mycological response	Partial response	Death (d114)	Partial response	Death (d85)	Progressive disease
Day 181 to 365 Clinical/radiological/mycological response	Complete mycological response, delayed wound healing (d365)	NA	Complete response (d270)	NA	Progressive disease (change of prosthesis planned d210)
Rezafungin related adverse event	No	No	No	No	No
Challenges and barriers to use of rezafungin	Cost coverage required prior to outpatient treatment	Stop of rezafungin after referral to other hospital (administrative hurdles)	Missing link of in‐hospital and outpatient setting	No, cost coverage clarified during in‐hospital stay	Administrative hurdles for OPAT approval, therefore CAS chosen, poor vein status

	Patient 6	Patient 7	Patient 8	Patient 9	Patient 10
Age (years)	64	44	77	57	73
Sex	M	F	M	M	M
Country	Italy	Italy	Italy	Italy	Italy
Date of invasive fungal infection diagnosis	Jun 23	Feb 24	Sep 24	Oct 24	Nov 24
Medical history	Type A aortic dissection 2012, recurrent *C. glabrata* candidemia 2015, 2016, replacement of ascending aorta with endoprosthesis 2016	CAD, CABG, obesity (BMI 30.2 kg/m2), intermittent haemodialysis	Aortic valve replacement, aortic aneurysm with abdominal aortic endoprosthesis, DM	Early prosthetic aortic valve endocarditis with MRSE, complicated by perivalvular abscess and mediastinitis	Rectal adenocarcinoma under radiochemotherapy, low anterior resection of the rectum
Fungal species	*C. glabrata*	*C. parapsilosis*	*C. albicans and C. krusei*	*C. parapsilosis*	*C. glabrata*
Diagnosis	Recurrent ascending aortic graft infection	Sternal osteomyelitis and mediastinitis	Abdominal aortic graft infection	Sternal wound infection, mediastinitis and possible prosthetic aortic endocarditis	Surgical site infection of intra‐abdominal seroma and peritonitis, and candidemia
MIC (μg/mL)	AND 0.06 FLU 64	AND 0.5 FLU 128	*C. albicans* : AND NA FLU S *C. krusei* : AND 0.06 FLU NA	AND 0.5 FLU 64	AND 0.03 FLU 4
Indication for rezafungin	OPAT, progressive vascular graft infection under VRC, DDI warfarin and VRC	OPAT (first two weeks in hospital administration), azole resistance	OPAT, *C. krusei*	OPAT, azole resistance, biofilm activity, conservative management	OPAT
Prior antifungal treatment and duration	CAS 30 d (2015), CAS 42 d (2023) VRC 54 weeks	CAS 28 d, AND 39 d	AND 45 d	AND 15 d	FLU 4 d, AND 12 d
Duration of rezafungin (weeks)	24	18	10	30 (ongoing)	4
D30 Clinical/radiological/mycological response	Complete response	Partial response	Complete response	Partial response	Partial response
Day 31 to 180 Clinical/radiological/mycological response	Complete response	Partial response	Complete response (d180)	Partial response (PET‐CT, d180)	Complete response (d62), alive d180
Day 181 to 365 Clinical/radiological/mycological response	Alive	Partial response until end of antifungal treatment, death due to intestinal perforation (d306)	NA	Alive d270, follow‐up PET‐CT planned	NA
Rezafungin‐related adverse event	No	No	Yes, mild cutaneous photosensitivity	No	No
Challenges and barriers to use of rezafungin	No	No	Per patient evaluation and approval by in‐hospital therapeutic committee	No	Per patient evaluation and approval by in‐hospital therapeutic committee

	Patient 11	Patient 12	Patient 13	Patient 14	Patient 15
Age (years)	63	67	68	34	56
Sex	F	F	F	M	M
Country	Italy	Italy	Spain	USA	Germany
Date of invasive fungal infection diagnosis	Jan 25	Jan 25	Nov 24	Apr 24	Nov 17
Medical history	DM, urolithiasis, lithotripsy, depressive syndrome	Kidney transplant with acute graft rejection, intermittent haemodialysis	Type A aortic dissection in Feb 2024, aortic endoprosthesis and biological aortic valve prosthesis	Schizophrenia with self‐harm, placement of ostomy, methamphetamine abuse, total parenteral nutrition	*Mycobacterium avium* infection 2009
Fungal species	*C. glabrata*	*C. glabrata*	*C. parapsilosis*	*C. auris*	*Aspergillus fumigatus*
Diagnosis	Candidemia following lithotripsy	Candidemia and intra‐abdominal candidiasis	Prosthetic aortic endocarditis, aortic graft infection, mycotic pseudoaneurysm right external carotid artery	Candidemia	Chronic cavernous pulmonary aspergillosis
MIC (μg/mL)	AND 0.03 FLU 4	AND 0.03 FLU 8	AND 1 FLU 0.5	AND NA FLU NA ISA < 0.3 MCF 0.25 VRC 1	AND 0.016 AMB 0.5 ISA 4 ITZ 2 VRC 2
Indication for rezafungin	OPAT, DDI sertraline and FLU	OPAT, elevated *C. glabrata* fluconazole MIC with concern of undertreatment in the context of haemodialysis	OPAT, echinocandin for vascular graft infection, conservative management	Worsening of liver function tests and skin rash under MCF	OPAT, insufficient VRC trough levels, VRC resistance, progressive nodular lesions
Prior antifungal treatment and duration	AND 9 d	CAS 14 d, FLU 20 d	FLU 77 d in combination with: AND 10 d, LAMB 7 d (acute kidney injury), AND 10 d (lack of supply), MCF 50 d	MCF 3d	VRC 2021–2024 (intermittent application), CAS 14d
Duration of rezafungin (weeks)	1	4	23 (ongoing)	2	18
D30 Clinical/radiological/mycological response	Complete response	Complete response	Partial response	Complete response	Partial response
Day 31 to 180 Clinical/radiological/mycological response	Complete response (d42)	Complete response	Partial response	Recurrence of *C. auris* candidemia (d60)	Complete response (d120)
Day 181 to 365 Clinical/radiological/mycological response	NA	NA	Partial response (d210)	NA	Complete response (d280)
Rezafungin‐related adverse event	No	No	No	No	Possible, mild fatigue and nausea, probably due to CPA symptoms
Challenges and barriers to use of rezafungin	Per patient evaluation and approval by in‐hospital therapeutic committee	Per patient evaluation and approval by in‐hospital therapeutic committee	Yes, in‐hospital administration request for approval	No, formulary‐listed; received on day of recommendation	NA: Off‐label use

*Note:* Day 0 refers to the day of diagnosis of the ongoing invasive fungal infection episode.

Abbreviations: AND, anidulafungin; *C*., *Candida*; CABG, coronary artery bypass grafting; CAD, coronary artery disease; d, days; DDI, drug–drug interaction; DM, diabetes mellitus; F, female; FLU, fluconazole; ISA, isavuconazole; ITZ, itraconazole; M, male; MCF, micafungin; MIC, minimum inhibitory concentration; NA, not available; OPAT, outpatient parenteral antimicrobial therapy; PET‐CT, positron emission tomography–computed tomography; S, susceptible; TAVI, transcatheter aortic valve implantation; VRC, voriconazole.

### 3.1 Patient 1

A 67‐year‐old male with coronary artery disease and type 2 diabetes mellitus developed a *C. glabrata* and *Streptococcus mitis* sternal osteomyelitis two weeks following coronary artery bypass grafting. Initial antifungal treatment with caspofungin was administered for two weeks, after which therapy was transitioned to rezafungin to facilitate hospital discharge and enable outpatient management. Although surgical removal of the sternal cerclage was indicated due to delayed wound closure, the patient refused the procedure. Three months after rezafungin treatment initiation, a surgical revision was performed, and persistence of *C. glabrata* was noted in the wound sampling. The cerclage remained in situ. Rezafungin was administered for a total duration of 36 weeks. At one‐year follow‐up, the patient was alive without signs of recurrent osteomyelitis, though delayed wound healing persisted, likely attributable to the retained cerclage.

### 3.2 Patient 2

A 79‐year‐old male with hepatitis B‐related liver cirrhosis (Child‐Pugh B) and chronic obstructive pulmonary disease (COPD) developed a *C. albicans* bloodstream infection, which was complicated by thoracic (T7–T10) spondylodiscitis and sensory‐motor paraplegia below T9 (biopsy‐proven *C. albicans* spondylodiscitis). Initial treatment with caspofungin was started. Given the underlying liver disease and the need for prolonged outpatient therapy, treatment was switched to rezafungin one month later. However, rezafungin treatment was limited to a single dose, as the patient was transferred to a secondary care hospital where rezafungin was not continued. Caspofungin was resumed at a reduced dose and pursued for 85 days. The patient succumbed to nosocomial pneumonia and hepatic decompensation.

### 3.3 Patient 3

A 55‐year‐old female with coronary artery disease and type 2 diabetes mellitus developed a prolonged *C. glabrata* candidemia of 13 days following coronary artery bypass grafting, treated for 26 days with caspofungin.

Three months later, she was diagnosed with a *C. glabrata* and *Staphylococcus caprae* sternal osteomyelitis. Due to the need for long‐term outpatient treatment of fluconazole‐resistant *C. glabrata* (fluconazole MIC 128 μg/mL), initial caspofungin therapy administered for 33 days was switched to rezafungin to enable outpatient management.

Although OPAT was intended, an outpatient referral was not arranged at discharge, resulting in a 44‐day interruption of the antifungal therapy. One month after reinitiating rezafungin, persistent growth of *C. glabrata* was documented in deep wound swabs obtained due to a draining wound. Computed tomography (CT) imaging five months into rezafungin treatment showed ongoing signs of infection. However, without the need for surgical revision, complete wound closure was eventually achieved after seven months. Follow‐up CT imaging four months thereafter showed full resolution of the infection. Rezafungin therapy was therefore discontinued after a total duration of nine months.

### 3.4 Patient 4

A 70‐year‐old male with coronary artery disease and uncontrolled diabetes mellitus was diagnosed with a *C. glabrata* candidemia treated with anidulafungin after transfemoral aortic valve replacement. Since there were no signs of endocarditis, treatment was stopped after 23 days. Eight months later, the patient was diagnosed with a *C. glabrata* aortic prosthetic valve endocarditis. Treatment with caspofungin was started, and due to high surgical risk, no operative intervention was performed. Given the absence of curative options and lack of viable oral suppressive therapies, rezafungin was initiated after 44 days of caspofungin treatment to facilitate outpatient care. Rezafungin was administered in the outpatient clinic, with plans to transition to full home‐based OPAT. Candidemia recurred multiple times, with positive blood cultures last noted 43 days after the start of rezafungin. Despite persistent infection, rezafungin was well tolerated, with no significant safety concerns reported. After 68 days from diagnosis, the patient died from cardiogenic shock due to ST‐elevation myocardial infarction.

### 3.5 Patient 5

A 58‐year‐old female with chronic mandibular osteomyelitis developed a left temporomandibular abscess and joint prosthesis infection caused by *C. glabrata* , *Enterobacter cloacae* , and *Pseudomonas aeruginosa* . Initial management included surgical drainage and caspofungin for 22 days, along with antibiotics. Rezafungin was introduced on day 27 as part of a conservative outpatient strategy alongside oral ciprofloxacin; however, only one dose was administered due to clinical deterioration requiring surgery, which revealed vancomycin‐resistant *Enterococcus faecium* and *E. faecalis* . Caspofungin and daptomycin were continued as OPAT for 10 weeks. Caspofungin was chosen over rezafungin as lower administrative hurdles for approval were expected. Despite therapy, disease progression was evident after 10 weeks, with a necrotic retroauricular wound and persistent *C. glabrata* positivity on day 75. Therefore, a change of the prosthesis is planned.

### 3.6 Patient 6

A 64‐year‐old male with a history of type A aortic dissection and aortic prosthesis placement in 2012 developed recurrent *C. glabrata* candidemia in 2015 and 2016 associated with vascular graft infection and consequently underwent replacement of the aortic prosthesis in 2016. In 2023, recurrence of candidemia was confirmed, with PET‐CT and intraoperative cultures indicating infection of the ascending aortic prosthesis. After 54 weeks of voriconazole suppressive therapy with adequate trough levels, but a *C. glabrata* MIC for voriconazole at 0.25 μg/mL (possibly resistant according to EUCAST clinical breakpoints), infection progressed, requiring the replacement of the ascending aorta and arch. Due to drug–drug interactions with warfarin, antifungal treatment was switched to rezafungin. Treatment with rezafungin was continued as OPAT for 24 weeks. PET‐CT four months after the end of antifungal treatment indicated sustained radiological response. The patient is alive two years after diagnosis, with no evidence of infection recurrence.

### 3.7 Patient 7

A 44‐year‐old female with coronary artery disease, obesity, and end‐stage chronic kidney disease on intermittent haemodialysis developed *C. parapsilosis* sternal osteomyelitis and mediastinitis following coronary artery bypass grafting. She initially received caspofungin (days 4–32) and anidulafungin (days 33–72) for progressive mediastinal collections in parallel with multiple wound revisions and sternal debridement. Due to fluconazole‐resistant *C. parapsilosis* (MIC 128 μg/mL), rezafungin was initiated on day 72 and continued until day 200 as OPAT. However, clinical and mycological responses remained incomplete despite long‐term antifungal treatment and multiple surgical interventions, with persistent abnormalities on PET‐CT and fluid collections. Due to clinical deterioration, antifungal therapy was reinitiated with anidulafungin (days 228–243), followed by liposomal amphotericin B (days 243–306). Cultures of fluid collections remained positive for *C. parapsilosis* at days 240 and 253. Despite all efforts, the patient died on day 306 due to intestinal perforation.

### 3.8 Patient 8

A 77‐year‐old male with a history of mechanical aortic valve replacement, subrenal aortic aneurysm treated with endovascular prosthesis, and uncontrolled diabetes mellitus was diagnosed with mixed candidemia with *C. albicans* and *Candida krusei* (*Pichia kudriavzevii*) related to an infection of a vascular endoprosthesis. Cultures were positive for *C. albicans* and *C. krusei* in the prosthesis and the surrounding tissue. Anidulafungin was initiated, and on day 12, the infected aortic endoprosthesis was surgically removed and replaced with a bovine pericardium graft. Anidulafungin was continued for a total duration of 45 days and then switched to rezafungin as OPAT for 10 weeks. The patient showed a partial clinical response by day 84 and PET‐CT confirmed resolution of tracer hyperaccumulation on the prosthesis by the end of rezafungin treatment at week 17 from diagnosis. Mild cutaneous photosensitivity occurred during rezafungin treatment but was managed conservatively without any treatment interruption. Treatment was otherwise well tolerated. At the six‐month follow‐up after diagnosis, the patient did not show any signs of recurrence.

### 3.9 Patient 9

**FIGURE 2:**
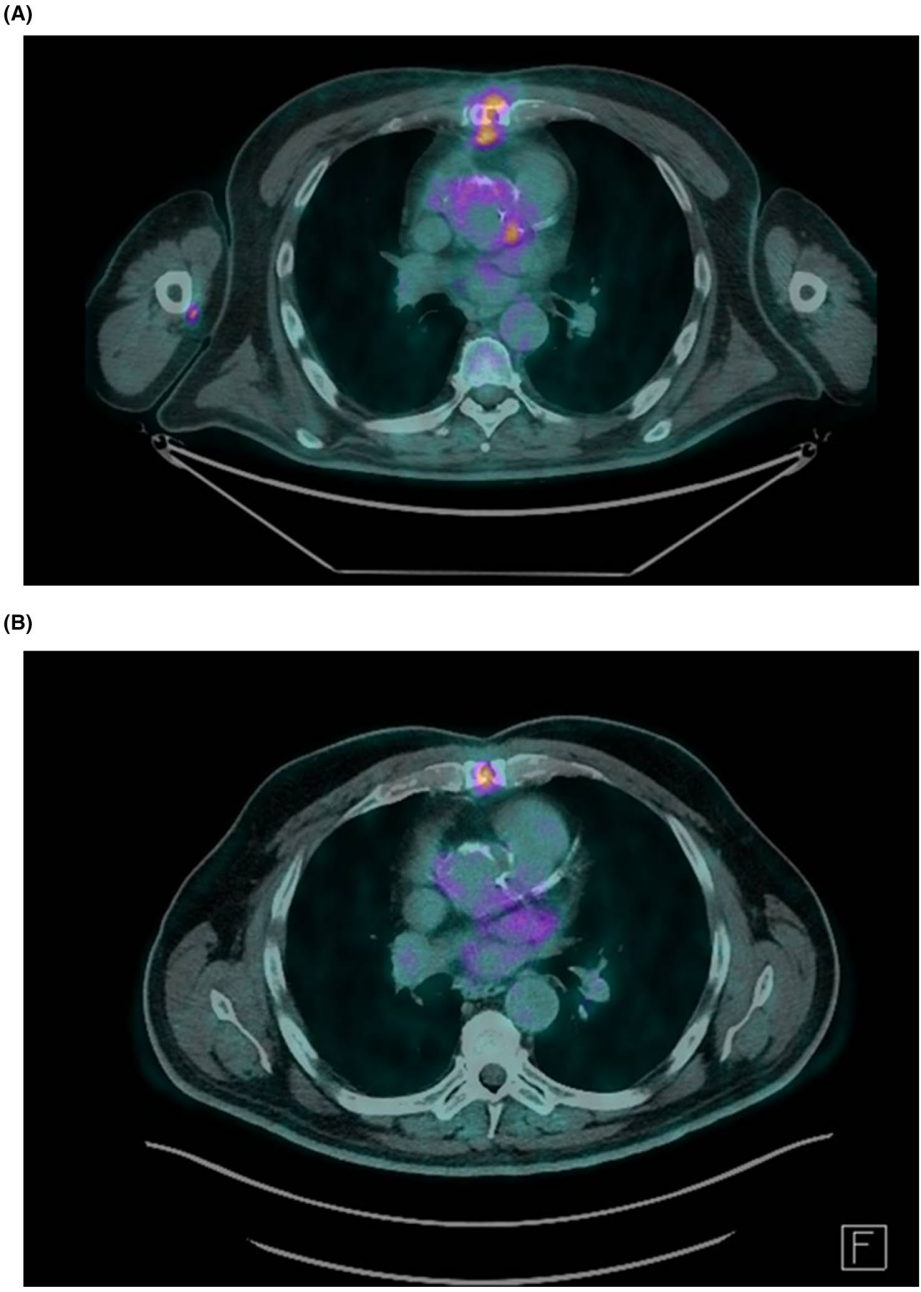
(A) Patient 9, initial PET‐CT scan showing increased tracer uptake (standardised uptake value, SUVmax 6) around the aortic root and in the anterior mediastinal fat (SUVmax 5), with hypermetabolic lymph nodes in the paratracheal and precarinal regions (SUVmax 5). Clinically, a sternal wound infection, mediastinitis and possible prosthetic aortic endocarditis with fluconazole‐resistant *C. parapsilosis* were concluded. (B) Patient 9, follow‐up PET‐CT 5.5 months later, after 23 weeks of rezafungin treatment, showing minimal heterogeneous tracer uptake (SUVmax 3.7) in the periaortic hypodense area and anterior mediastinal fat consolidation. Moderate uptake remains at the median sternotomy site. Compared to the prior PET‐CT, findings are reduced in extent and metabolic activity. The next PET‐CT is scheduled 3 months later; the patient remains under rezafungin until then.

A 57‐year‐old male with early prosthetic aortic valve endocarditis caused by methicillin‐resistant *Staphylococcus epidermidis* , complicated by perivalvular abscess, underwent aortic valve replacement with a bioprosthesis, valved conduit implantation, and coronary vein bypass. He was diagnosed nine days later with *C. parapsilosis* sternal wound infection, mediastinitis, and possible prosthetic valve endocarditis. Initial antifungal therapy with anidulafungin for 15 days was followed by rezafungin due to *C. parapsilosis* resistance to fluconazole (MIC 64 μg/mL) and the need for biofilm‐active treatment. Six months after diagnosis, a partial response on PET‐CT was documented (Figure 2). A follow‐up imaging is scheduled in two months. The patient remains clinically stable and alive at 9 months following diagnosis, with rezafungin planned to continue until complete PET‐CT resolution.

### 3.10 Patient 10

**FIGURE 3:**
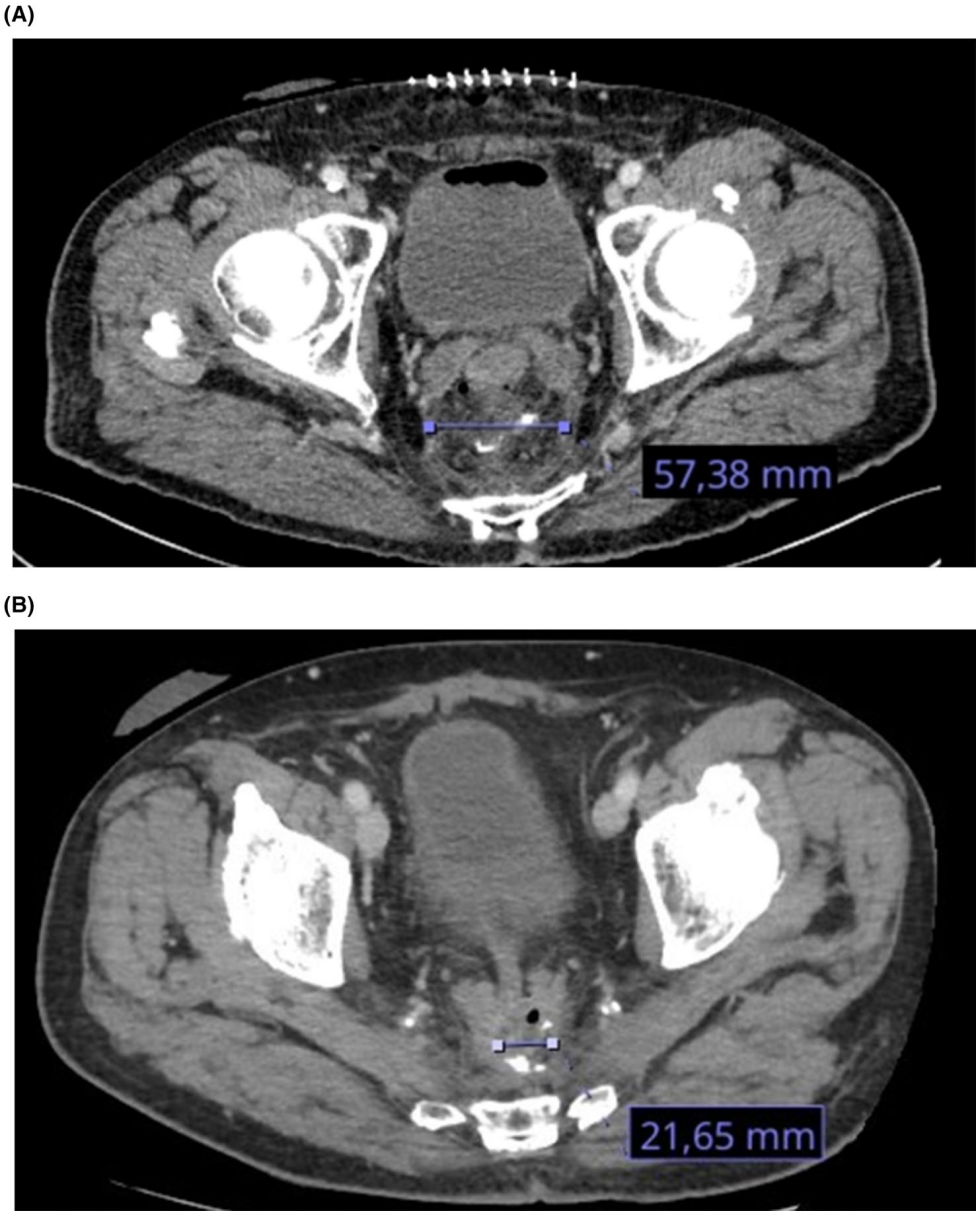
(A) Patient 10, initial CT scan on postoperative day five after low anterior resection of the rectum, showing a seroma of 57 mm. Culture of the collection fluid grew *C. glabrata* . (B) Patient 10, follow‐up CT scan on postoperative day 38 after three weeks of rezafungin therapy, showing a reduction of the seroma to 21 mm. Surgical intervention was not pursued, as the collection was self‐draining into the rectal stump.

A 73‐year‐old male with rectal adenocarcinoma underwent radiochemotherapy and low anterior resection of the rectum. He developed *C. glabrata* candidemia (MIC fluconazole 4 μg/mL) and intra‐abdominal candidiasis with infected seroma and peritonitis after surgery. Initial antifungal therapy with fluconazole was switched to anidulafungin after four days due to disease progression. Given the need for outpatient management and persistent infection, rezafungin was initiated on day 12 and administered weekly for five weeks. Source control was not surgically pursued, as the collection was self‐draining into the rectal stump. Clinical improvement included resolution of fever and rectal discharge and radiological reduction of the collection by day 33 (cf. Figure 3). Candidemia cleared by day 10, with partial mycological response by day 28 and 1,3‐ß‐D‐Glucan normalisation by day 41. At the six‐month follow‐up, the patient remained alive, without clinical or radiological signs of recurrence.

### 3.11 Patient 11

A 63‐year‐old female with a history of urolithiasis treated with lithotripsy and urethral stent placement, uncontrolled diabetes mellitus, and a depressive disorder developed *C. glabrata* candidemia following lithotripsy. Initial antifungal therapy with anidulafungin was administered for nine days, with blood cultures clearing by day 3. Fluconazole MIC was 4 μg/mL. Due to a potential drug–drug interaction between fluconazole and concurrent sertraline therapy, rezafungin was selected for OPAT. A single dose of rezafungin was administered on day 9, with the patient maintaining a sustained clinical response through day 42 of treatment.

### 3.12 Patient 12

A 67‐year‐old female kidney transplant recipient with antibody‐mediated acute graft rejection and uncontrolled diabetes mellitus developed *C. glabrata* candidemia and intra‐abdominal candidiasis six weeks after kidney transplantation. Peritoneal cultures were positive for *C. glabrata* for fluid collections in the right iliac fossa and communicating with supra‐fascial tissues in the right flank. Initial antifungal therapy included 14 days of caspofungin followed by 20 days of fluconazole. Due to a fluconazole MIC of 8 μg/mL and concerns of undertreatment during intermittent dialysis, therapy was switched to rezafungin as OPAT on day 34. After four doses, treatment was discontinued due to CT‐confirmed significant reduction of intra‐abdominal collections. Candidemia cleared by day 12 (under caspofungin), and 1,3‐ß‐D‐Glucan levels normalised by day 52 (under rezafungin). The patient did not show any signs or symptoms of recurrence on day 90.

### 3.13 Patient 13

A 68‐year‐old female with type A aortic dissection underwent placement of an aortic endoprosthesis and a biological aortic valve prosthesis. Nine months later, she developed *C. parapsilosis* candidemia, complicated by aortic endoprosthesis infection and a mycotic pseudoaneurysm of the right external carotid artery confirmed by PET‐CT. Initial antifungal therapy included fluconazole with concurrent start of anidulafungin, followed by liposomal amphotericin B (discontinued due to acute kidney injury), followed by a prolonged course of micafungin (50 days). Due to high surgical risk, no operative intervention was performed. Rezafungin was initiated on day 77 for OPAT, and fluconazole was continued. Clinical response has been partial, and although decreasing, 1,3‐ß‐D‐Glucan remained positive six months after diagnosis. Fluconazole is planned as lifelong suppression, whereas treatment with rezafungin was continued according to the PET‐CT performed seven months after initial diagnosis, which showed no change from the prior PET‐CT.

### 3.14 Patient 14

A 34‐year‐old incarcerated male with schizophrenia and repetitive self‐harm leading to the placement of an ostomy and total parenteral nutrition via peripherally inserted central catheter (PICC) line developed a *C. albicans* candidemia, treated with micafungin and PICC replacement. He was readmitted two months later with a *C. auris* candidemia. Prior antifungal treatment with micafungin was discontinued due to elevated liver enzymes and a new rash. Rezafungin was initiated on day 4, with blood cultures cleared the same day. Rezafungin was well tolerated. Treatment was stopped after 18 days. However, *C. auris* candidemia recurred two months after treatment completion and was managed with a 3‐week course of micafungin without any occurrence of drug‐related adverse events.

### 3.15 Patient 15

A 56‐year‐old male with a history of *Mycobacterium avium* infection treated in 2009 and subsequent diagnosis of chronic pulmonary aspergillosis (CPA) with *Aspergillus fumigatus* in 2017 was treated with intermittent voriconazole from 2021 to 2024. Despite dose adjustments, voriconazole trough levels remained subtherapeutic and progression of the disease was observed on CT imaging in the left upper lobe. This progression prompted a left apical partial pneumectomy. Voriconazole therapy was continued due to uncertainty regarding the complete resection of the infection. However, the identification of azole resistance prompted the off‐label use of rezafungin rather than the daily application of amphotericin B as OPAT. Following a two‐week bridging course of caspofungin administered in an in‐hospital setting, rezafungin was initiated three months after surgery. Rezafungin treatment was continued for 18 weeks as OPAT. The patient reported mild fatigue and nausea following infusions, which resolved spontaneously. *Aspergillus* IgG declined under rezafungin from initially 178 mgA/L (norm: < 50 mgA/L) to 158 mgA/L by the end of treatment. During therapy, clinical improvement was observed, and the patient experienced improved exercise tolerance and resolution of cough. Therefore, treatment was discontinued by the patient. Follow‐up thoracic CT five months after rezafungin discontinuation showed no signs of aspergillosis. Six months after discontinuation of rezafungin, *Aspergillus* antibody titre was above 200 mgA/L. Since fungal cultures and PCR from sputum remained negative and the patient showed no clinical or radiological signs (CT scan) of recurrence, no antifungal treatment was initiated.

## 4 Results

### 4.1 Clinical Characteristics of Rezafungin‐Treated Patients

Between February 2024 and May 2025, fifteen patients who received rezafungin were identified in Germany (*n* = 6), Italy (*n* = 7), Spain (*n* = 1), and the United States (*n* = 1). Fourteen patients were diagnosed with IC. One patient with CPA by *A. fumigatus* received rezafungin in an off‐label setting and was therefore excluded from the following analysis.

Median age was 65.5 years (mean: 62.6 years, range: 34–79 years) with eight (57%) male and six (43%) female patients. The most frequent underlying conditions were coronary artery disease (4/14, 29%), diabetes mellitus (5/14, 36%), or immunosuppression due to malignancy or solid organ transplantation (2/14, 14%).

*C. glabrata* (8/14, 57%) was the most frequently identified fungal species, followed by *C. parapsilosis* (3/14, 21%). One patient had a mixed candidemia with *C. albicans* and *C. krusei* . In two out of eight patients, *C. glabrata* MIC for fluconazole was higher than the clinical breakpoint of 16 μg/mL and classified as resistant according to EUCAST [12].

Primary indications for long‐term antifungal use were intravascular infections (5/14, 36%) and osteoarticular infections (5/14, 36%). In all 14 patients, rezafungin was started after prior treatment with other echinocandins, in most patients caspofungin (8/14, 57%) or anidulafungin (6/14, 43%).

Rezafungin was started to allow for outpatient management in 12 patients (86%). Rationale for selecting rezafungin as therapy rather than azoles included its biofilm activity (7/14, 50%), treatment of fluconazole‐resistant isolates (4/14, 29%), and anticipated drug–drug interactions (2/14, 14%) (Figure 1). Amongst these patients, rezafungin was administered as part of conservative management in five patients, as they were considered not eligible to undergo surgery.

Median rezafungin treatment duration was 9 weeks (mean 14.3 weeks, range: 1 to 38 weeks). In 8 patients (57%) treatment duration was more than 4 weeks. Rezafungin was administered via peripheral venous access in all patients. Treatment was well tolerated in 13 patients (93%). One patient experienced an adverse event possibly related to rezafungin (cutaneous hypersensitivity). No severe drug‐related adverse event was reported.

At day 30 after IC diagnosis, five patients (36%) had a complete response and seven (50%) had a partial clinical, radiological, or mycological response. Disease progression was reported in two patients (14%). Outcomes beyond day 30 to day 365 is described in Table 1. Due to non‐homogeneous frequency of follow‐up, precise outcome data are limited to day 30. Death occurred in three patients (21%). One death could be directly attributed to IC (patient 4). The other two succumbed to complications resulting from their underlying medical conditions.

### 4.2 Logistical and Regulatory Considerations of Rezafungin Application

In the three German centres, high acquisition costs of rezafungin required approval from health insurance companies before patient discharge. This process frequently delayed or hindered access to rezafungin and contributed to longer hospital stays, consequently resulting in increased overall treatment costs. In two patients, treatment with rezafungin was ultimately not pursued due to logistical challenges and therapy was continued with caspofungin (patients 2 and 5). Rezafungin gained the status of a new examination and treatment method “Neue Untersuchungs‐ und Behandlungsmethoden” (NUB) in 2024. Since receiving NUB status 1, acquisition costs for rezafungin are covered by health insurance; however, each hospital is still required to negotiate individual and yearly reimbursement agreements.

In the two Italian centres, access to rezafungin differed markedly. Rezafungin was classified as a class C(nn) drug by the Italian Medicines Agency (AIFA). In one centre, the absence of a formal pricing agreement between AIFA and the manufacturer necessitated patient‐by‐patient evaluation and approval by the hospital therapeutic committee, followed by submission of a purchase request to the manufacturer for each patient. Consequently, the delay from initial request to first administration of rezafungin in this centre was at least 2 to 3 weeks. In contrast, the other Italian centre reported no such barriers, with rezafungin obtained smoothly through the hospital pharmacy for OPAT and used in accordance with the product label.

In the Spanish centre, specific approval for use of rezafungin for treatment of the patient had to be requested from the hospital manager prior to patient discharge to enable the switch from micafungin to rezafungin.

In the US centre, the patient received rezafungin on the same day as recommended by the ID consultation, as it was included in the hospital's drug formulary. However, this was limited to in‐hospital administration only, without transition to OPAT.

## 5 Discussion

With the known broad‐spectrum activity and favourable tolerability of the echinocandin class, combined with rezafungin's novel pharmacokinetic profile enabling once‐weekly dosing, the drug has been anticipated to be a valuable asset in the treatment of difficult‐to‐manage and logistically complex fungal infections [13]. However, various regulatory, logistical, and administrative hurdles challenge its seamless integration into real‐life clinical practice. In this case series, we describe the clinical characteristics, treatment rationale, and outcomes of rezafungin use in 15 patients, alongside the access and cost approval barriers encountered by treating physicians across different healthcare settings.

In accordance with other reports, the most frequent rationale for rezafungin included OPAT and/or azole resistance [14]. Other situations favouring the use of an echinocandin over an azole were drug–drug interactions, rapid metabolism of voriconazole and anticipated biofilm activity in case of infections associated with foreign material [15, 16, 17]. In vitro, significantly higher doses of rezafungin were required to target mature biofilm compared to planktonic cells for *C. parapsilosis* and *C. auris* , while *C. albicans* required lower doses [18]. These findings were consistent with the effects observed for caspofungin in the same study. Although limited to in vitro findings, future clinical research may evaluate the administration of higher rezafungin doses in settings involving mature biofilms, as studied and recommended for caspofungin [2, 19, 20, 21, 22]. In the phase 2 trial, a four‐week course of rezafungin at 400 mg weekly did not raise any safety concerns compared to patients receiving 200 mg weekly [8]. The trial, however, excluded patients with osteoarticular candidiasis or endocarditis.

No dose modification is recommended in patients undergoing intermittent or continuous renal replacement therapy [23, 24]. In our cohort, two patients undergoing intermittent haemodialysis received rezafungin at standard dosages. Treatment resulted in one complete and one partial response. Partial response was associated with culture‐positive results for *C. parapsilosis* until day 253 (patient 7) and was attributed to incomplete source control despite repetitive surgical interventions. However, it again raises the question of individualised higher dosing strategies, particularly in patients with deep‐seated infections caused by *C. parapsilosis* . Rezafungin shows broad tissue distribution (kidney, lung, liver, spleen, heart) and superior tissue penetration compared to other echinocandins [6, 25]. Interestingly, the three patients with sternal osteomyelitis had mycological persistence. To this day, published clinical experience in osteoarticular infections is limited to case reports [26, 27, 28] and pharmacokinetic analysis in animal or human bone tissue is lacking.

An important setting noted was the use of rezafungin in the context of conservative management or palliative care, where patients were not eligible for indicated surgery. Although source control is crucial in the management of deep‐seated candidiasis, use of rezafungin may offer benefits in health‐related quality of life and symptom control. In these patients, rezafungin administration reduced the need for central venous line placement, minimising associated complications such as infections or thrombosis and reducing daily costs related to human resources [14]. In injection drug users, central venous lines may facilitate drug use and increase the risk of *Candida* endocarditis, which is often managed conservatively [29]. Rezafungin may offer a valuable treatment alternative to other echinocandins in this setting.

The case series highlights the use of rezafungin in intravascular and in osteoarticular infections, requiring prolonged treatment courses, particularly if foreign material remains in place [30, 31, 32]. Treatment durations exceeding four weeks showed no relevant safety or tolerability issues in any of the patients. This is consistent with findings reported [14, 28, 33]. Further conditions requiring long‐term antifungal therapy, such as hepatosplenic candidiasis (HSC), warrant greater consideration of rezafungin as a treatment option. HSC often presents as a breakthrough infection during azole prophylaxis, with *Candida* species frequently unidentified, making echinocandins the empirical first‐line therapy [2].

*C. glabrata* was the most frequent *Candida* species observed. Despite most of the patients presenting fluconazole MIC below the clinical breakpoint, echinocandins were selected by clinicians either due to the presence of biofilms or as MICs were near the clinical breakpoint and as fluconazole resistance can be rapidly induced [34, 35, 36, 37]. Candidemia due to *C. auris* was successfully treated with rezafungin, and recurrence of candidemia was likely related to remaining risk factors for *C. auris* and candidemia, as the PICC line remained in place [38]. The role of rezafungin in treating *Candida* infections with FKS mutations, an emerging global threat in IC, remains to be elucidated in future studies [39, 40, 41, 42].

In the patient with voriconazole‐resistant CPA, complete symptom control was achieved after four months of rezafungin as an off‐label application and prior pneumonectomy. Successful application of rezafungin in a patient with CPA was previously described [14]. In the upcoming phase 2 multicentre single‐arm study enrolling adult patients with CPA, the efficacy of rezafungin will be evaluated in the setting of limited treatment options [43].

As a newly available antifungal agent, rezafungin remains unfamiliar to many clinicians, particularly in primary and secondary care settings. Administrative and bureaucratic processes may limit current routine use. Certain obstacles outlined in Table 2 are not unique to rezafungin but represent common challenges of new drugs until reimbursement pathways and clinical experience are established. Pending cost approvals may favour other echinocandins to avoid reimbursement uncertainty, as seen in two patients switched from rezafungin to caspofungin. Despite advantages such as once‐weekly dosing and reduced demand on healthcare personnel, rezafungin is rarely considered as a treatment option during hospitalisation. However, health‐economic modelling suggests that early outpatient treatment with rezafungin may reduce overall treatment costs compared to a prolonged in‐hospital stay [14, 46]. Notably, many of the reported patients had IC after surgery, while rezafungin is relatively unknown to surgical disciplines. This underscores the importance of an infectious disease or clinical microbiology consultation service to recommend the best available therapy for each patient. Finally, a meticulously organised transition from in‐ to outpatient care setting and OPAT program is crucial to limit unwanted treatment interruptions.

**TABLE 2:** Considerations on troubleshooting of rezafungin application.

Obstacle/Problem	Possible causes	Possible solutions
Limited awareness of rezafungin as a treatment option	Lack of ID physician/clinical microbiologist availability or consultation, absence of multidisciplinary case discussion	Online board for mycological case discussion with broad access to all types of health institutions^a^, enhance in‐hospital multidisciplinary discussion
Hesitation to prescribe rezafungin	Administrative hurdles, complex healthcare administration, pending cost approvals risking missing cost coverage	Structured pathways, fast‐tracked approvals (e.g., if rezafungin prescribed by ID physician), introduce support system for hospital administration/clinicians in prescription process
High acquisition and overall treatment costs, health‐economic burden	Novel antifungal treatment	Enhance health‐economic analysis, ensure indication accuracy, shorten hospital stays
Treatment interruption during transition of care	Missing link between in‐ and outpatient care	Funding of OPAT programs, prior discharge: inpatient visit by OPAT team and facilitation of outpatient appointments [44]
Patient burden	Long travel distances to reach facilities capable of treatment application	Initiate rezafungin prior hospital discharge, training and support of out‐of‐hospital nurses or primary care personnel (limitation: application takes over one hour)

*Note:* Summary of barriers to rezafungin application are not country‐specific and reflect possible centre‐specific obstacles (e.g., in‐ to outpatient care transition) and individual reimbursement challenges.

^a^ Following the example of Ref. [45].

This case series is limited by its retrospective design, small sample size, and absence of a control group, all of which constrain the generalisability of the findings. Moreover, the clinical impact of rezafungin is difficult to isolate due to multiple confounding factors, including surgical interventions and underlying comorbidities. Future studies with larger sample sizes could yield more robust findings, particularly in subgroup and outcome analyses.

Given its multiple advantages, along with demonstrated efficacy and safety in this and other real‐world observations, rezafungin should be integrated into clinical practice as a standard treatment option—particularly for deep‐seated candidiasis and long‐term therapy. Reducing existing barriers to prescription and administration could facilitate its broader adoption.

## Author Contributions

**Ilana Reinhold:** conceptualization, writing – original draft, formal analysis, visualization, investigation. **Giovanni Mori:** writing – review and editing. **Massimiliano Lanzafame:** writing – review and editing. **Alessandro Limongelli:** writing – review and editing. **Antonio Vena:** writing – review and editing. **Julia Götz:** writing – review and editing. **Stilla Bauernfeind:** writing – review and editing. **Frank Hanses:** writing – review and editing. **Lukas Tometten:** writing – review and editing. **Michael Mayer:** writing – review and editing. **Ansgar Rieke:** writing – review and editing. **Ana Soriano‐Martin:** writing – review and editing. **Maricela Valerio:** writing – review and editing. **Jose A. Vazquez:** writing – review and editing. **Patrick Yue:** writing – review and editing. **Laman Rahimli:** writing – review and editing, investigation. **Nijat Azimli:** investigation. **Ertan Sal:** investigation. **Jon Salmanton‐García:** writing – review and editing, investigation. **Natalia Vasenda:** investigation. **Rosanne Sprute:** writing – review and editing. **Jannik Stemler:** writing – review and editing. **Sebastian Wingen‐Heimann:** writing – review and editing. **Oliver A. Cornely:** conceptualization, writing – original draft. **Danila Seidel:** conceptualization, writing – original draft, methodology, formal analysis, visualization, investigation.

## Conflicts of Interest

J.S. has received research funding by the Ministry of Education and Research (BMBF), the Medical Faculty of the University of Cologne, Basilea Pharmaceutica, Noscendo, Scynexis; has received speaker honoraria by AbbVie, Akademie für Infektionsmedizin, FoMF, Gilead, Hikma, Lilly and Pfizer; has served on advisory boards for Kite‐Gilead; has been a consultant to Gilead, Mundipharma, Alvea Vax and Micron Research.

R.S. reports grants from the German Center for Infection Research (DZIF Clinical Leave) and the Ministry of Culture and Science of the State of North Rhine‐Westphalia (FF‐Med), lecture and speaker honoraria from Akademie für Infektionsmedizin e.V., Ärztliche Akademie für medizinische Fort‐ und Weiterbildung in Nordrhein, FomF GmbH, Infektio Saar Netz, Hikma, Mundipharma and Pfizer and travel support from the ECMM, ESCMID, ISHAM, Page Medical and Pfizer; all outside of the submitted work.

J.G. has received speaker honoraria from Akademie für Infektionsmedizin, Ärztliche Akademie für medizinische Fort‐ und Weiterbildung and FoMF and travel support from Gilead.

J.S.‐G. has received payment or honoraria for lectures, presentations, speakers bureaus, manuscript writing, or educational events from Gilead, Menarini, and Pfizer; and has participated on a Data Safety Monitoring Board or Advisory Board for Pfizer, outside of the submitted work.

O.A.C. reports grants or contracts from iMi, iHi, DFG, BMBF, Cidara, DZIF, EU‐DG RTD, F2G, Gilead, MedPace, MSD, Mundipharma, Octapharma, Pfizer, Scynexis; Consulting fees from Abbvie, AiCuris, Basilea, Biocon, Boston Strategic Partners, Cidara, Elion Therapeutics, Gilead, GSK, IQVIA, Janssen, Matinas, MedPace, Menarini, Melinta, Molecular Partners, MSG‐ERC, Mundipharma, Noxxon, Octapharma, Pardes, Pfizer, PSI, Scynexis, Seres, Seqirus, Shionogi, The Prime Meridian Group; Speaker and lecture honoraria from Abbott, Abbvie, Akademie für Infektionsmedizin, Al‐Jazeera Pharmaceuticals/Hikma, amedes, AstraZeneca, Deutscher Ärzteverlag, Gilead, GSK, Grupo Biotoscana/United Medical/Knight, InfectoPharm, Ipsen Pharma, Medscape/WebMD, MedUpdate, MSD, Moderna, Mundipharma, Noscendo, Paul‐Martini‐Stiftung, Pfizer, Sandoz, Seqirus, Shionogi, streamedup!, Touch Independent, Vitis; Payment for expert testimony Cidara; Participation on a DRC, DSMB, DMC, Advisory Board for AstraZeneca, Cidara, IQVIA, Janssen, MedPace, Melinta, PSI, Pulmocide, Vedanta Biosciences.

All other authors do not declare any conflicts of interest related to this manuscript.

## Data Availability

The data that support the findings of this study are available from the corresponding author upon reasonable request.
